# miR-663 promotes NPC cell proliferation by directly targeting CDKN2A

**DOI:** 10.3892/mmr.2021.12539

**Published:** 2021-11-22

**Authors:** Shaoqiang Liang, Ning Zhang, Yanming Deng, Lusi Chen, Yang Zhang, Zhenhe Zheng, Weijun Luo, Zhiqian Lv, Shaoen Li, Tao Xu

Mol Med Rep 16: 4863-4870, 2017; DOI: 10.3892/mmr.2017.7129

Following the publication of this paper, it was drawn to the Editors’ attention by a concerned reader that certain of the cell cycle assay data shown in Figs. 2D and 5C were strikingly similar to data appearing in different form in other articles by different authors. Owing to the fact that the contentious data in the above article had already been published elsewhere, or were already under consideration for publication, prior to its submission to *Molecular Medicine Reports*, the Editor has decided that this paper should be retracted from the Journal. After having been in contact with the authors, they agreed with the decision to retract the paper. The Editor apologizes to the readership for any inconvenience caused.

